# Understanding the combining ability of nutritional, agronomic and industrial traits in soybean F_2_ progenies

**DOI:** 10.1038/s41598-023-45271-4

**Published:** 2023-10-20

**Authors:** Paulo Henrique Menezes das Chagas, Larissa Pereira Ribeiro Teodoro, Dthenifer Cordeiro Santana, Marcelo Carvalho Minhoto Teixeira Filho, Paulo Carteri Coradi, Francisco Eduardo Torres, Leonardo Lopes Bhering, Paulo Eduardo Teodoro

**Affiliations:** 1 Department of Agronomy, State University of São Paulo (UNESP), Ilha Solteira, SP 15385-000 Brazil; 2 Federal University of Mato Grosso do Sul (UFMS), Chapadão do Sul, MS 79560-000 Brazil; 3 Department of Agricultural Engineering, Federal University of Santa Maria, Cachoeira do Sul, RS 96503-205 Brazil; 4 State University of Mato Grosso do Sul (UEMS), Aquidauana, MS 79200-000 Brazil; 5 Federal University of Viçosa (UFV), Viçosa, MG 36570-900 Brazil

**Keywords:** Plant sciences, Environmental sciences

## Abstract

Obtaining soybean genotypes that combine better nutrient uptake, higher oil and protein levels in the grains, and high grain yield is one of the major challenges for current breeding programs. To avoid the development of unpromising populations, selecting parents for crossbreeding is a crucial step in the breeding pipeline. Therefore, our objective was to estimate the combining ability of soybean cultivars based on the F_2_ generation, aiming to identify superior segregating parents and populations for agronomic, nutritional and industrial traits. Field experiments were carried out in two locations in the 2020/2021 crop season. Leaf contents of the following nutrients were evaluated: phosphorus, potassium, calcium, magnesium, sulfur, copper, iron, manganese, and zinc. Agronomic traits assessed were days to maturity (DM) and grain yield (GY), while the industrial traits protein, oil, fiber and ash contents were also measured in the populations studied. There was a significant genotype × environment (G × A) interaction for all nutritional traits, except for P content, DM and all industrial traits. The parent G3 and the segregating populations P20 and P27 can be used aiming to obtain higher nutritional efficiency in new soybean cultivars. The segregating populations P11 and P26 show higher potential for selecting soybean genotypes that combine earliness and higher grain yield. The parent G5 and segregant population P6 are promising for selection seeking improvement of industrial traits in soybean.

## Introduction

Soybean (*Glycine max* L.) is one of the most important crops in the world due to its high grain yield and protein and oil contents. The increased production over the last decades is due to technological progress and genetic breeding targeting the improvement of several traits, such as increased grain yields^1^ and obtaining genotypes adapted to the abiotic conditions of the growing region^2^. Thus, genetic variability is essential to breeding programs, accounting for the feasibility of techniques to identify superior genotypes and use them to generate improved soybean cultivars^3^. There is undeniable an effort in the breeding programs to select genotypes with higher yields than those already grown today. However, some gaps also need to be addressed by breeders, especially regarding traits of interest to the industry (oil and protein contents)^4^, uptake of nutrients by genotypes in Brazilian Cerrado^5^, besides seeking to increase grain yields^6^.

Developing soybean varieties that combine high grain yield and satisfactory oil and protein contents is a complex process due to the crop cycle and the genetic grain characteristics, which are highly influenced by environmental factors^7^. However, throughout the soybean breeding program, it is possible to select genotypes for higher oil and protein contents up to a given threshold, after which the selection for higher means for one variable results in the decrease of the another^8^. Such an association between contents of oil, protein and other variables of industrial interest has been reported in the literature. Jiang et al.^9^ grain fiber content throughout the breeding processes. Conversely, Santana et al.^10^, in a study to classify soybean genotypes for industrial variables, they found a strong positive correlation between oil and protein contents.

Given this scenario, the need for further studies also investigating the factors involved in the expression of these traits, such as inheritance and gene effects controlling the trait, and genetic variability among genotypes is evident. Such information is crucial for obtaining improved genotypes for industrial variables. Among the approaches that can help to solve these shortcomings is the study of the combining ability between genotypes through diallel crossings. This approach provides parameter estimates that are useful for selecting parents for hybridization and understanding the action of genes involved in determining the traits evaluated^11^. When selecting parents, diversity and performance per se are relevant due to the possibility of planning crossings between parents or groups of parents that provide a high heterotic effect in their offspring, making it possible to obtain populations with a broad genetic variety^12^.

Diallel studies carried out to establish segregating populations in soybean predominantly evaluate agronomic traits (plant height, cycle, grain yield, among others). Research seeking to understand the inheritance of agronomic traits together with nutritional (macro and micronutrient contents) and industrial traits (such as protein and oil contents) in soybean is scarce. The objectives were: (i) to select parents and segregating populations for nutritional, agronomic, and industrial traits in soybean and (ii) to understand the relationship between these traits.

## Results

### Joint analysis of variance and grouping of means for nutritional traits

There was a significant effect of genotypes (p-value < 0.05) for the nutritional contents of P and Mg (Table S1). GCA effect was significant only for Mg content, while SCA effects were significant for P and Mg content. Environment effects (E) were significant for all nutrient contents, except for K. Genotypes by environments interaction (G × E) and SCA × E were significant for all nutritional contents, except P. GCA × E interaction was significant for K, Ca, Fe, Mn, and Zn.

Table 1 shows the grouping of means among the genotypes for the levels of macronutrients evaluated. Segregating population P1 stood out by presenting the highest mean levels of K, Ca, Mg, and S in all sites. The populations P4 and P5 stood out by obtaining the highest means of P, K, Ca, and S in both environments. Except for the Mg content in Chapadão do Sul, the population P20 and the parent G7 had the highest averages for all macro-nutrients evaluated.

**Table 1:** Grouping of means for the nutritional contents of macronutrients (P, K, Ca, Mg and S) evaluated in parents and F_2_ segregating populations of soybean in Aquidauana (E1) and Chapadão do Sul (E2).

Genotype	P (g kg^−1^)	K (g kg^−1^)		Ca (g kg^−1^)		Mg (g kg^−1^)		S (g kg^−1^)	
		E1	E2	E1	E2	E1	E2	E1	E2
G1	3.02 a	17.38 a	13.84 b	9.56 a	11.47 a	4.62 a	3.76 b	2.60 b	1.90 a
P1	2.87 b	16.45 a	18.12 a	8.45 a	12.37 a	4.35 b	4.61 a	3.11 a	1.81 a
P2	2.90 b	18.17 a	16.49 a	8.02 a	8.27 b	4.79 a	3.12 c	3.40 a	1.79 a
P3	2.52 b	17.84 a	16.89 a	7.76 a	7.81 b	4.08 b	2.83 d	2.79 b	1.74 a
P4	3.19 a	17.65 a	17.32 a	7.32 a	10.85 a	4.42 b	3.19 c	3.15 a	2.37 a
P5	3.27 a	16.88 a	16.99 a	7.05 a	10.70 a	3.90 c	3.22 c	3.33 a	2.10 a
P6	2.95 b	17.38 a	17.05 a	6.70 a	8.62 b	4.20 b	3.14 c	3.34 a	1.84 a
P7	2.97 b	17.43 a	11.61 b	8.00 a	12.46 a	4.40 b	4.04 b	3.18 a	2.18 a
G2	2.78 b	14.37 b	16.67 a	6.02 a	6.87 b	4.40 b	3.88 b	2.35 b	1.88 a
P8	2.89 b	17.27 a	15.86 a	9.77 a	10.99 a	4.49 b	2.77 d	2.59 b	1.73 a
P9	2.69 b	15.70 b	14.53 b	7.48 a	7.85 b	4.21 b	2.49 d	2.83 b	1.81 a
P10	2.91 b	17.29 a	17.60 a	7.50 a	9.38 b	4.26 b	3.27 c	3.38 a	1.72 a
P11	3.14 a	15.23 b	16.47 a	7.23 a	9.54 b	4.13 b	2.83 d	2.73 b	1.96 a
P12	2.99 b	13.22 b	19.04 a	5.30 a	9.30 b	3.43 c	3.00 c	2.37 b	2.12 a
P13	2.94 b	15.47 b	16.99 a	6.74 a	12.46 a	4.15 b	3.36 c	3.00 a	1.91 a
G3	2.97 b	18.15 a	18.08 a	7.63 a	8.80 b	4.76 a	3.75 b	2.94 a	1.82 a
P14	3.12 a	16.97 a	15.22 b	7.28 a	9.81 b	3.47 c	2.72 d	3.22 a	1.69 a
P15	2.89 b	15.59 b	17.14 a	7.10 a	11.03 a	4.17 b	2.72 d	2.92 b	1.79 a
P16	2.94 b	12.00 b	15.99 a	4.98 a	11.09 a	3.39 c	3.40 c	2.72 b	1.85 a
P17	2.90 b	15.56 b	14.54 b	8.05 a	9.66 b	4.49 b	4.19 b	2.79 b	2.18 a
P18	3.36 a	14.37 b	18.61 a	7.01 a	13.09 a	3.69 c	3.55 c	2.81 b	2.41 a
G4	2.92 b	13.53 b	13.05 b	6.12 a	3.70 d	3.22 c	2.39 d	2.73 b	1.79 a
P19	3.24 a	17.36 a	13.72 b	8.02 a	10.68 a	4.37 b	3.89 b	2.61 b	2.06 a
P20	3.13 a	19.15 a	17.73 a	8.33 a	9.68 b	4.88 a	3.49 c	3.11 a	1.65 a
P21	2.86 b	15.42 b	14.26 b	8.03 a	8.83 b	4.88 a	3.23 c	2.58 b	1.83 a
P22	2.97 b	12.45 b	16.12 a	5.71 a	8.34 b	3.42 c	3.36 c	2.81 b	2.31 a
G5	3.19 a	12.77 b	17.42 a	7.13 a	12.76 a	3.63 c	3.47 c	3.02 a	2.26 a
P23	2.82 b	13.43 b	11.74 b	8.15 a	11.28 a	4.13 b	3.90 b	2.56 b	1.95 a
P24	3.23 a	15.76 b	13.93 b	7.65 a	9.09 b	4.62 a	3.24 c	2.98 a	1.89 a
P25	2.92 b	15.76 b	16.06 a	8.23 a	8.49 b	3.29 c	2.25 d	2.85 b	1.98 a
G6	2.83 b	14.73 b	15.95 a	6.56 a	6.57 c	3.88 c	2.38 d	2.80 b	1.95 a
P26	2.92 b	12.89 b	17.49 a	7.34 a	6.23 c	2.90 c	3.40 c	2.56 b	1.92 a
P27	3.07 a	18.58 a	15.06 b	7.72 a	8.37 b	4.74 a	4.93 a	2.86 b	1.97 a
G7	3.36 a	21.83 a	17.01 a	7.95 a	10.69 a	5.55 a	3.45 c	3.26 a	1.60 a
P28	2.70 b	15.45 b	13.47 b	6.84 a	9.49 b	4.29 b	3.52 c	3.03 a	1.65 a
G8	2.94 b	17.43 a	12.63 b	7.15 a	9.83 b	4.35 b	3.02 c	3.26 a	1.72 a

Means followed by different letters in the same column differ by the Scott Knott test at 5% probability.

The grouping of means for the micronutrient contents are shown in Table 2. The segregant population P7 stood out by obtaining higher means for Cu and Zn in Aquidauana and Fe, Mn, and Zn in Chapadão do Sul. The population P21 stood out by having higher Cu and Mn means in Aquidauana and Fe in Chapadão do Sul.

**Table 2:** Grouping of means for the nutritional contents of micronutrients (Cu, Fe, Mn e Zn) evaluated in parents and F_2_ segregating populations of soybean in Aquidauana (E1) and Chapadão do Sul (E2).

Genotype	Cu (mg kg^−1^)		Fe (mg kg^−1^)		Mn (mg kg^−1^)		Zn (mg kg^−1^)	
	E1	E2	E1	E2	E1	E2	E1	E2
G1	13.00 a	16.00 b	330.50 c	112.50 a	74.00 c	32.50 b	54.50 b	49.00 b
P1	13.00 a	19.50 a	361.00 b	108.50 a	71.00 c	30.50 b	54.00 b	27.00 c
P2	12.00 a	20.50 a	133.00 e	106.50 a	79.00 b	27.00 b	71.00 a	36.00 c
P3	8.50 a	7.50 c	303.00 c	103.50 a	83.00 b	28.50 b	64.50 a	30.50 c
P4	11.50 a	10.00 c	122.00 e	115.50 a	78.50 b	39.50 a	74.00 a	40.50 b
P5	12.00 a	4.00 c	99.50 e	99.50 a	76.00 c	26.50 b	63.00 a	31.50 c
P6	10.00 a	6.50 c	190.00 d	94.50 a	69.00 c	27.50 b	67.00 a	29.50 c
P7	12.00 a	15.50 b	150.00 e	108.00 a	82.00 b	39.50 a	71.50 a	60.00 a
G2	10.00 a	3.50 c	163.00 e	106.50 a	72.50 c	28.50 b	64.50 a	37.00 c
P8	9.00 a	5.00 c	306.00 c	103.00 a	83.00 b	30.50 b	51.50 b	39.50 b
P9	14.00 a	12.50 b	125.50 e	97.50 a	74.00 c	35.00 a	57.50 b	44.50 b
P10	12.50 a	10.00 b	308.00 c	111.50 a	76.00 c	27.50 b	59.50 b	30.50 c
P11	10.00 a	9.50 c	131.00 e	103.00 a	80.00 b	29.50 b	61.50 b	30.50 c
P12	11.00 a	11.00 b	179.00 d	106.00 a	80.50 b	28.50 b	65.00 a	27.50 c
P13	11.50 a	11.50 b	143.50 e	109.50 a	80.00 b	33.00 b	70.00 a	32.00 c
G3	16.50 a	15.00 b	123.00 e	113.00 a	93.00 a	29.00 b	64.50 a	36.00 c
P14	11.50 a	10.00 c	130.00 e	108.50 a	75.00 c	33.00 b	64.00 a	44.50 b
P15	16.50 a	13.00 b	147.00 e	195.00 a	72.50 c	31.50 b	60.50 b	42.00 b
P16	11.00 a	15.50 b	119.00 e	110.50 a	81.50 b	39.50 a	68.00 a	26.50 c
P17	11.00 a	12.00 b	122.00 e	111.50 a	77.00 c	28.00 b	65.00 a	30.00 c
P18	10.50 a	6.00 c	137.00 e	107.00 a	75.50 c	31.00 b	52.50 b	33.00 c
G4	11.00 a	7.00 c	404.00 b	109.50 a	68.00 c	28.50 b	53.00 b	35.00 c
P19	16.00 a	14.00 b	102.50 e	105.00 a	86.00 b	30.00 b	62.00 b	29.50 c
P20	11.00 a	7.50 c	369.00 b	101.50 a	89.00 a	31.00 b	75.00 a	32.00 c
P21	14.00 a	13.50 b	655.50 a	102.50 a	92.50 a	29.00 b	50.50 b	36.00 c
P22	11.50 a	10.50 c	137.50 e	116.00 a	83.50 b	30.00 b	70.50 a	45.00 b
G5	11.00 a	3.50 c	114.50 e	100.00 a	68.50 c	31.50 b	51.50 b	25.50 c
P23	11.50 a	18.00 a	113.50 e	102.00 a	44.50 d	42.00 a	53.50 b	26.50 c
P24	12.00 a	12.00 b	116.50 e	96.00 a	70.50 c	29.50 b	61.00 b	33.00 c
P25	9.50 a	20.00 a	231.50 d	121.50 a	74.50 c	28.50 b	61.00 b	36.00 c
G6	10.00 a	12.50 b	134.50 e	116.00 a	72.50 c	30.50 b	54.50 b	42.00 b
P26	17.00 a	13.00 b	149.00 e	117.50 a	64.50 c	43.00 a	55.00 b	30.50 c
P27	10.50 a	6.00 c	229.50 d	124.50 a	76.50 c	32.00 b	59.00 b	56.50 a
G7	11.00 a	11.00 b	380.00 b	121.50 a	81.00 b	26.50 b	77.00 a	31.50 c
P28	11.50 a	3.00 c	107.50 e	103.00 a	69.00 c	35.00 a	66.50 a	33.00 c
G8	12.00 a	7.50 c	424.00 b	110.00 a	83.00 b	27.50 b	71.00 a	44.00 b

Means followed by different letters in the same column differ by the Scott Knott test at 5% probability.

### Joint analysis of variance and grouping of means for agronomic traits

There were significant effects of genotypes and SCC (p-value < 0.05) for grain yield (Table S2). Environmental effects (E) were significant for DM and GY. G × E, GCA × E and SCA × E interactions were significant for DM.

Table 3 contains the grouping of means for the agronomic traits DM and GY. The G1 parent showed the highest mean for DM in Aquidauana. The segregant populations P1, P5, P16, P19 and parents G2 and G7 showed higher means for DM in both sites. Segregant populations P3, P10, P18, P21, P22, P24, P28 and parents G5, G6 and G8 obtained the highest DM means in Chapadão do Sul. Segregant populations P6, P7, P11, P17, P20, P26 and P27 obtained higher means for GY.

**Table 3:** Grouping of means for the agronomic traits days to maturity and grain yield (kg ha^−1^) evaluated in parents and F_2_ segregating populations of soybean in Aquidauana (E1) and Chapadão do Sul (E2).

Genotype	DM		GY (kg ha^−1^)
	E1	E2	
G1	104.50 a	103.50 b	2488.05 b
P1	106.50 a	104.50 a	2892.19 b
P2	98.50 d	101.50 b	2646.08 b
P3	97.00 d	107.00 a	2606.03 b
P4	102.50 b	103.00 b	2521.83 b
P5	107.50 a	107.00 a	2698.79 b
P6	96.50 d	108.00 a	4508.14 a
P7	101.00 c	106.00 a	3491.30 a
G2	105.50 a	104.50 a	2167.32 b
P8	101.00 c	103.00 b	1942.32 b
P9	102.00 b	102.50 b	2195.95 b
P10	103.00 b	104.50 a	2760.15 b
P11	97.50 d	101.00 b	3594.34 a
P12	100.00 c	103.00 b	2737.19 b
P13	103.00 b	103.00 b	2633.44 b
G3	99.50 c	101.50 b	2388.21 b
P14	100.00 c	102.00 b	2313.24 b
P15	103.50 b	103.00 b	3111.25 b
P16	104.50 a	106.00 a	3017.77 b
P17	102.00 b	104.50 a	3545.05 a
P18	96.50 d	104.50 a	2425.13 b
G4	102.00 b	103.00 b	2979.56 b
P19	107.00 a	104.50 a	2793.22 b
P20	105.00 a	103.00 b	4497.47 a
P21	100.00 c	106.00 a	2598.00 b
P22	103.50 b	104.50 a	3170.48 b
G5	104.00 b	104.50 a	2724.62 b
P23	101.00 c	103.00 b	2515.58 b
P24	103.50 b	106.00 a	2789.39 b
P25	102.50 b	103.00 b	2733.54 b
G6	101.50 b	104.50 a	2735.35 b
P26	98.00 d	104.50 a	3895.00 a
P27	103.00 b	107.00 a	3466.19 a
G7	105.00 a	105.50 a	1739.59 b
P28	103.00 b	104.50 a	3023.57 b
G8	103.50 b	104.50 a	2016.76 b

Means followed by different letters in the same column differ by the Scott Knott test at 5% probability.

### Analysis of variance and grouping of means for industrial traits

There was significant effects of genotypes (G) and environments (E) (p-value < 0.05) for the industrial traits PC and OC (Table S3). There were significant GCA and SCA effects for OC. G × E and SCA × E interaction were significant for all the evaluated traits. GCA × E interaction was significant for all traits except AC.

Table 4 contains the grouping of means for the industrial traits. The segregant population P16 obtained the highest means for PC in Chapadão do Sul, OC and FC in both sites. The parents G2 and G5 and segregant populations P10, P15 and P20 obtained higher means for PC in both sites. The segregant population P24 obtained higher means for FC in both environments. Populations P13 and P17 obtained higher AC in both environments evaluated.

**Table 4:** Grouping of means for the industrial traits protein (PC), oil (OC), fiber (FC) and ash (AC) contents assessed in leaf samples from parents and F_2_ segregating populations of soybean in Aquidauana (E1) and Chapadão do Sul (E2).

Genotype	PC		OC		FC		AC	
	E1	E2	E1	E2	E1	E2	E1	E2
G1	37.24 c	37.81 a	21.07 d	20.23 e	5.48 c	5.37 c	5.10 b	4.96 a
P1	37.24 c	37.24 a	20.17 e	20.17 e	5.62 c	5.62 c	4.96 c	4.96 a
P2	36.73 c	36.20 a	21.82 c	21.83 c	5.48 c	5.31 c	5.02 c	4.91 a
P3	37.00 c	36.48 a	21.27 d	21.66 c	5.61 c	5.27 b	5.13 b	4.94 a
P4	37.87 b	36.35 a	21.28 d	21.63 c	5.42 c	5.85 c	5.16 b	4.98 a
P5	37.98 c	36.79 a	21.30 d	20.80 d	5.36 c	5.52 c	4.94 c	4.89 a
P6	36.31 c	36.31 a	20.85 d	20.85 d	5.32 c	5.32 c	4.93 c	4.93 a
P7	36.46 c	36.14 a	20.80 d	21.53 c	6.16 a	5.38 c	5.17 b	4.91 a
G2	38.64 a	36.82 a	20.38 d	21.03 d	5.46 c	5.16 c	5.16 b	4.92 a
P8	37.53 b	37.23 a	21.55 d	21.28 d	5.44 c	5.26 c	4.87 c	4.99 a
P9	38.75 a	35.73 b	21.58 d	21.86 c	5.83 b	5.63 c	5.07 b	4.92 a
P10	39.31 a	36.60 a	20.13 e	20.35 e	5.49 c	5.46 c	5.04 c	5.03 a
P11	36.35 c	36.35 a	21.19 d	21.19 d	5.67 c	5.67 b	4.96 c	4.96 a
P12	36.59 c	36.59 a	20.86 d	20.86 d	5.55 c	5.55 c	4.98 c	4.98 a
P13	37.99 b	36.62 a	20.28 e	21.56 c	5.55 c	5.40 c	5.30 a	4.97 a
G3	35.01 d	35.01 b	21.97 c	21.97 c	5.51 c	5.51 c	4.88 c	4.88 a
P14	38.96 a	34.91 b	20.64 e	22.42 b	4.93 d	5.67 b	4.83 c	4.93 a
P15	38.69 a	36.28 a	21.49 c	21.64 c	5.72 c	5.39 c	5.11 b	4.97 a
P16	36.40 c	36.40 a	23.15 a	23.12 a	6.20 a	6.10 a	4.93 c	4.93 a
P17	38.43 a	35.29 b	20.69 e	22.55 b	4.89 d	5.40 c	5.30 a	4.96 a
P18	37.59 b	35.69 b	21.67 c	21.46 c	5.81 b	5.30 c	5.03 c	4.95 a
G4	37.71 b	35.05 b	20.47 e	21.88 c	5.36 c	5.75 b	4.88 c	4.93 a
P19	36.58 c	36.58 b	21.87 c	21.87 c	5.23 d	5.23 c	4.97 c	4.97 a
P20	38.39 a	36.07 a	20.56 e	21.54 c	5.39 c	5.37 c	4.90 c	4.96 a
P21	37.73 b	37.47 a	20.33 e	21.38 c	5.85 b	5.28 c	5.12 b	4.94 a
P22	38.79 a	35.93 b	20.68 e	20.84 d	4.78 d	6.07 a	4.97 c	4.96 a
G5	38.73 a	37.12 a	20.65 e	21.49 c	5.54 c	5.35 c	5.05 b	4.96 a
P23	37.81 b	35.81 b	22.20 c	22.42 b	5.51 c	5.56 c	5.12 b	4.96 a
P24	36.30 c	36.30 a	20.92 d	20.92 d	6.15 a	6.15 a	4.94 c	4.94 a
P25	37.02 c	36.33 a	22.43 b	22.19 b	5.59 c	5.30 c	5.04 c	4.90 a
G6	35.60 d	35.60 b	20.51 e	20.51 e	5.80 b	5.80 b	4.97 c	4.97 a
P26	39.43 a	35.50 b	20.88 d	21.82 c	4.81 d	5.48 c	5.11 b	4.91 a
P27	37.08 c	36.54 a	21.46 c	21.78 c	5.59 c	5.28 c	4.93 c	4.94 a
G7	35.54 d	35.54 b	21.23 d	21.23 d	5.46 c	5.46 c	4.98 c	4.98 a
P28	36.70 c	36.00 b	22.14 c	22.41 b	5.68 c	5.25 c	4.98 c	4.98 a
G8	35.35 d	35.35 b	21.98 c	21.98 c	5.60 c	5.60 c	4.98 c	4.98 a

Means followed by different letters in the same column differ by the Scott Knott test at 5% probability.

### Combining ability for nutritional traits

The G3 parent stood out for contributing positive values to the contents of K, Mg, Ca, Fe, Mn, and Zn in both evaluated environments (Table 5), except for Fe levels in Aquidauana and Mn in Chapadão do Sul, whose values were negative. Similarly, G8 presented positive estimates for K, Mg, Ca, Fe, Mn, and Zn in both locations, except for K levels in Chapadão do Sul and Ca in Aquidauana, which showed negative values.

**Table 5:** Estimates of the general combining (gi effect) ability for nutritional contents of K, Mg, Ca, Fe, Mn, and Zn, evaluated in soybean parents in Aquidauana (E1) and Chapadão do Sul (E2).

Parent	K (g kg^−1^)		Mg	Ca (g kg^−1^)		Fe (mg kg^−1^)		Mn (mg kg^−1^)		Zn (mg kg^−1^)	
	E1	E2		E2	E1	E2	E1	E2	E1	E2	E1
G1	1.23	− 0.05	0.18	0.6	0.74	14.65	− 3.34	− 0.26	0.14	1.29	2.96
G2	− 0.49	0.93	0.02	− 0.2	− 0.1	0.7	− 4.24	0.04	− 1.11	− 1.31	− 1.79
G3	0.20	0.73	0.03	0.1	0.49	− 53.3	7.36	4.04	− 0.41	0.04	0.01
G4	− 0.23	− 0.81	− 0.24	− 0.16	− 1.62	75.8	− 4.09	2.99	− 0.91	− 1.11	0.86
G5	− 0.59	− 0.03	− 0.08	0.18	0.97	− 50.3	5.21	− 4.96	0.89	− 2.66	− 3.44
G6	− 0.66	0.07	− 0.08	− 0.26	− 0.66	− 39.35	− 0.39	− 3.21	2.19	− 1.71	− 0.54
G7	0.51	0.11	0.16	− 0.07	− 0.4	40.65	− 2.04	− 0.41	− 0.91	2.29	− 4.09
G8	0.01	− 0.95	0.02	− 0.19	0.58	11.15	1.51	1.79	0.14	3.19	6.01

SCA estimates for macronutrient contents evaluated in the F_2_ segregating populations of soybean are shown in Table 6. Population P7 stood out for presenting positive estimates for the contents of P, K, Ca, Mg and S at both locations, except for the K levels in Chapadão do Sul. Similarly, P20 also presented positive estimates for these traits, except for S contents in Chapadão do Sul.

**Table 6:** Estimates of specific combining ability (sij effects) for macronutrient contents (P, K, Ca, Mg, and S), evaluated in F_2_ segregating populations of soybean in Aquidauana (E1) and Chapadão do Sul (E2).

Population	P (g kg^−1^)	K (mg kg^−1^)		Ca (mg kg^−1^)		Mg (mg kg^−1^)		S (mg kg^−1^)	
		E1	E2	E1	E2	E1	E2	E1	E2
P1	− 0.02	− 0.32	1.39	0.67	2.1	− 0.04	1.1	0.27	− 0.1
P2	− 0.08	0.71	− 0.04	− 0.06	− 2.59	0.38	− 0.38	0.4	− 0.15
P3	− 0.41	0.81	1.9	− 0.06	− 0.93	− 0.1	− 0.36	− 0.12	− 0.15
P4	0.15	0.98	1.55	− 0.84	− 0.49	0.16	− 0.26	0.13	0.32
P5	0.29	0.28	1.12	− 0.68	0.99	− 0.29	− 0.28	− 0.22	0.14
P6	− 0.06	− 0.39	1.14	− 1.22	− 1.34	− 0.4	− 0.43	0.35	− 0.05
P7	0.01	0.16	− 3.24	0.21	1.52	0.13	0.43	0.1	0.17
P8	− 0.02	1.53	− 1.65	2.48	0.97	0.24	− 0.58	− 0.21	− 0.12
P9	− 0.16	0.39	− 1.44	0.45	− 0.05	0.19	− 0.54	0.13	0
P10	− 0.07	2.34	0.85	0.13	− 1.12	0.16	− 0.02	0.57	− 0.25
P11	0.23	0.35	− 0.38	0.3	0.67	0.1	− 0.51	0.09	0.08
P12	0.05	− 2.83	2.15	− 1.82	0.18	− 1.01	− 0.41	− 0.41	0.31
P13	0.05	− 0.08	1.16	− 0.26	2.36	0.03	− 0.09	0.13	− 0.02
P14	0.17	0.97	− 0.56	− 0.04	1.32	− 0.57	− 0.3	0.35	− 0.15
P15	− 0.17	− 0.05	0.59	− 0.56	− 0.06	0.05	− 0.56	− 0.05	− 0.21
P16	− 0.07	− 3.57	− 0.67	− 2.25	1.63	− 0.66	0.07	− 0.08	− 0.05
P17	− 0.14	− 1.18	− 2.16	0.63	− 0.06	0.03	0.79	− 0.15	0.35
P18	0.37	− 1.87	2.97	− 0.28	2.4	− 0.45	0.11	− 0.22	0.45
P19	0.22	2.15	− 1.29	0.62	1.71	0.48	0.93	− 0.27	0.1
P20	0.18	4.01	2.62	1.36	2.34	1.05	0.48	0.40	− 0.21
P21	− 0.12	− 0.89	− 0.9	0.87	1.23	0.65	0.15	− 0.27	0.04
P22	0.04	− 3.36	2.03	− 1.32	− 0.24	− 0.49	0.24	− 0.13	0.39
P23	− 0.25	− 1.35	− 4.15	0.84	1.34	0.23	0.63	− 0.26	− 0.07
P24	0.13	− 0.19	− 2.00	0.15	− 1.1	0.31	− 0.1	0.02	− 0.07
P25	− 0.14	0.31	1.19	0.86	− 2.68	− 0.7	− 1.13	− 0.2	− 0.1
P26	− 0.12	− 2.99	1.46	0.28	− 2.33	− 1.34	0.01	− 0.23	0.06
P27	0.08	3.20	0.09	0.78	− 1.17	0.82	1.49	− 0.02	− 0.01
P28	− 0.32	− 1.1	− 1.55	− 0.29	− 0.31	− 0.04	0.01	0.02	− 0.26

Table 7 contains CEC estimates for micronutrient levels evaluated in F_2_ segregating populations. Population P7 stood out for presenting positive estimates for Cu, Mn, and Zn levels at both locations. Population P13 presented positive estimates for all micronutrients except for Fe in Aquidauana and Zn in Chapadão do Sul, which were negative. Population P15 presented positive estimates for all micronutrients at both locations except for Mn.

**Table 7:** Estimates of specific combining ability (sij effects) for micronutrient contents (Cu, Fe, Mn e Zn), evaluated in F_2_ segregating populations of soybean in Aquidauana (E1) and Chapadão do Sul (E2).

Population	Cu (mg kg^−1^)		Fe (mg kg^−1^)		Mn (mg kg^−1^)		Zn (mg kg^−1^)	
	E1	E2	E1	E2	E1	E2	E1	E2
P1	1.88	8.08	137.54	5.59	− 5.34	0.07	− 8.32	− 10.11
P2	− 0.47	6.48	− 36.46	− 8.01	− 1.34	− 4.13	7.33	− 2.91
P3	− 3.37	− 4.27	4.44	0.44	3.71	− 2.13	1.98	− 9.26
P4	− 0.67	− 3.22	− 50.46	3.14	7.16	7.07	13.03	5.04
P5	0.68	− 8.67	− 83.91	− 7.26	2.91	− 7.23	1.08	− 6.86
P6	− 1.87	− 5.62	− 73.41	− 10.61	− 6.89	− 3.13	1.08	− 5.31
P7	0.88	3.93	− 83.91	0.66	3.91	7.82	4.68	15.09
P8	− 3.07	− 6.07	150.49	− 10.61	2.36	0.62	− 9.57	5.34
P9	2.53	3.68	− 159.11	− 4.66	− 5.59	5.62	− 2.42	9.49
P10	0.73	− 0.27	149.49	0.04	4.36	− 3.68	1.13	− 0.21
P11	− 0.92	− 0.22	− 38.46	− 2.86	6.61	− 2.98	2.18	− 3.11
P12	− 0.47	1.83	− 70.46	1.79	4.31	− 0.88	1.68	− 2.56
P13	0.78	2.88	− 76.46	1.74	1.61	2.57	5.78	− 8.16
P14	− 1.32	− 1.42	− 100.61	− 5.26	− 8.59	2.92	2.73	7.69
P15	3.38	0.13	42.49	71.94	− 3.14	− 0.38	0.78	9.49
P16	− 1.27	3.18	3.54	− 6.96	4.11	6.32	7.33	− 8.91
P17	− 1.82	0.23	− 73.46	− 4.31	− 3.19	− 2.08	0.33	− 1.86
P18	− 1.57	− 5.22	− 28.96	− 12.36	− 6.89	− 0.13	− 13.07	− 8.96
P19	3.48	3.38	− 131.11	− 6.61	11.41	− 1.38	3.43	− 3.86
P20	− 0.67	− 2.57	124.44	− 4.51	12.66	− 1.68	15.48	− 4.26
P21	1.78	3.98	330.94	− 1.86	13.36	− 0.58	− 13.02	3.29
P22	0.03	1.53	− 157.56	8.09	2.16	− 0.63	6.08	2.19
P23	− 0.47	6.48	− 4.96	− 13.31	− 23.89	7.52	− 4.47	− 5.46
P24	− 0.52	1.03	− 81.96	− 17.66	− 0.69	− 1.88	− 0.97	4.59
P25	− 2.27	9.58	62.54	4.29	1.11	− 3.93	− 1.87	− 2.51
P26	5.33	2.58	− 60.41	9.44	− 8.44	10.32	− 7.92	− 0.81
P27	− 0.42	− 3.87	49.59	12.89	1.36	− 1.73	− 4.82	15.09
P28	0.03	− 6.32	− 152.41	− 6.96	− 8.94	4.37	− 1.32	− 4.86

### Combining ability for agronomic traits

For the GCA of the DM trait (Table 8), the parent G3 stood out for presenting negative values at both evaluated locations. Conversely, the parents G6 and G8 presented positive GCA estimates for this trait in Aquidauana and Chapadão do Sul.

**Table 8:** Estimates of general combining ability (gi effects) for the days to maturity (DM) trait in soybean parents in Aquidauana (E1) and Chapadão do Sul (E2).

Parent	DM	
	E1	E2
G1	− 0.01	0.61
G2	0.54	− 0.74
G3	− 1.36	− 1.04
G4	− 0.01	− 0.24
G5	1.24	− 0.19
G6	0.09	0.26
G7	− 0.56	0.96
G8	0.09	0.36

The segregating populations P1 and P15 stood out by presenting positive SCC estimates for the traits days to maturity and grain yield (Table 9). Other populations that deserve to be highlighted are P11, P12 and P26 for presenting negative estimates for DM in both locations, as well as positive GCA estimates for grain yield.

**Table 9:** Specific combining ability estimates (sij effects) for agronomic traits days to maturity (DM) and grain yield (GY) evaluated in segregating F_2_ populations of soybean in Aquidauana (E1) and Chapadão do Sul (E2).

Population	DM		GY (kg ha^−1^)
	E1	E2	
P1	3.91	0.42	223.83
P2	− 2.19	− 2.28	− 90.98
P3	− 5.04	2.42	− 366.66
P4	− 0.79	− 1.63	− 304.92
P5	5.36	1.92	− 576.13
P6	− 4.99	2.22	1491.26
P7	− 1.14	0.82	634.25
P8	− 0.24	0.57	− 469.71
P9	− 0.59	− 0.73	− 451.71
P10	− 0.84	1.22	258.42
P11	− 5.19	− 2.73	644.45
P12	− 2.04	− 1.43	45.33
P13	0.31	− 0.83	101.42
P14	− 0.69	− 0.93	− 403.13
P15	1.56	0.02	540.82
P16	3.71	2.57	− 0.83
P17	1.86	0.37	784.49
P18	− 4.29	0.97	− 175.6
P19	3.71	0.72	− 12.84
P20	2.86	− 1.23	1243.25
P21	− 1.49	1.07	− 398.19
P22	1.36	0.17	334.13
P23	− 2.39	− 1.28	− 592.71
P24	0.76	1.02	− 60.86
P25	− 0.89	− 1.38	43.12
P26	− 3.59	− 0.93	596.58
P27	0.76	2.17	327.61
P28	1.41	− 1.03	143.01

### Combining ability for industrial traits

The parent G5 stood out by contributing positive values for all industrial traits in both locations (Table 10).

**Table 10:** General combining ability estimates (gi effects) for the industrial traits protein (PC), oil (OC) and fiber (FC) contents evaluated in soybean parents in Aquidauana (E1) and Chapadão do Sul (E2).

Parent	PC		OC		FC	
	E1	E2	E1	E2	E1	E2
G1	− 0.24	0.51	− 0.1	− 0.46	0.02	− 0.05
G2	0.46	0.40	− 0.41	− 0.42	0.04	− 0.06
G3	− 0.21	− 0.4	0.43	0.47	− 0.02	0.00
G4	0.52	− 0.27	− 0.27	0.18	− 0.14	0.05
G5	0.46	0.25	0.10	0.04	0.05	0.01
G6	− 0.18	− 0.14	0.12	0.02	0.04	0.11
G7	− 0.39	− 0.15	− 0.15	− 0.03	− 0.05	− 0.02
G8	− 0.41	− 0.21	0.28	0.22	0.07	− 0.04

Segregant populations P1, P24, P25, P26 and P27 stood out by showing positive estimates of protein content for both environments (Table 11). The segregant population P6 showed positive estimates for protein and oil content in Aquidauana and Chapadão do Sul. The population P11 obtained positive estimates for oil and fiber in both locations and ash in Aquidauana. The segregant population P19 obtained positive estimates for oil and ash contents in both locations evaluated.

**Table 11:** Specific combining ability estimates (sij effects) for the industrial traits protein (PC), oil (OC), fiber (FC), and ash (AC) contents evaluated in F_2_ segregating populations of soybean in Aquidauana (E1) and Chapadão do Sul (E2).

Population	PC		OC		FC		AC	
	E1	E2	E1	E2	E1	E2	E1	E2
P1	0.33	0.56	0.09	− 0.35	− 0.09	− 0.03	0.02	0.03
P2	− 0.36	0.11	− 0.5	− 0.45	0.04	0.23	− 0.12	0.01
P3	− 0.21	− 0.14	0.31	0.32	− 0.04	− 0.14	0	− 0.02
P4	− 0.66	0.02	0.46	0.44	0.2	− 0.23	0.12	0.01
P5	0.27	− 0.63	0.10	0.54	− 0.17	0.39	0.08	0.03
P6	1.02	0.19	0.10	0.26	− 0.23	− 0.05	− 0.08	− 0.04
P7	− 0.45	− 0.28	− 0.08	− 0.16	− 0.17	− 0.11	− 0.14	− 0.02
P8	− 0.27	− 0.39	− 0.56	0.27	0.55	− 0.04	0.1	− 0.03
P9	0.34	− 0.2	0.02	0.37	− 0.14	− 0.22	0.08	− 0.05
P10	− 0.1	1.01	0.35	− 0.27	− 0.1	− 0.18	− 0.15	0.04
P11	0.39	− 0.62	1.08	0.6	0.41	0.14	0.06	− 0.03
P12	1.01	− 0.27	− 0.74	− 0.78	− 0.12	0.01	− 0.04	0.06
P13	− 1.31	− 0.14	0.31	0.09	0.07	0.12	− 0.06	0.01
P14	− 0.86	0.11	0.24	− 0.19	0.05	0.13	− 0.08	0.01
P15	0.56	0.2	− 0.77	0.26	− 0.07	− 0.01	0.23	0.01
P16	− 1.95	− 0.42	− 0.08	− 0.48	0.03	0	− 0.07	− 0.04
P17	1.27	− 0.64	− 0.7	0.27	− 0.43	0.12	− 0.11	0
P18	1.06	0.21	− 0.22	− 0.38	0.17	− 0.13	0.1	0.02
P19	− 0.59	0.71	1.42	1.16	0.66	0.58	− 0.02	0
P20	1.65	− 0.39	− 0.78	0.6	− 0.56	− 0.09	0.3	0.02
P21	0.83	0.07	− 0.22	− 0.73	0.24	− 0.17	0.03	0.01
P22	− 0.71	− 0.63	− 0.16	0.02	0.11	0.15	− 0.05	− 0.01
P23	− 1.78	0.38	0.86	0.14	− 0.2	− 0.33	− 0.04	0.01
P24	0.67	0.26	− 0.46	− 0.16	− 0.04	− 0.3	− 0.04	0.02
P25	0.22	1.67	− 0.43	− 0.27	0.52	− 0.25	0.13	− 0.01
P26	1.3	0.19	− 0.51	− 1.06	− 0.67	0.55	− 0.03	0.01
P27	0.43	0.4	− 0.73	− 0.1	− 0.08	− 0.17	− 0.03	− 0.02
P28	0.15	− 0.53	0.81	0.85	− 0.1	− 0.07	0.11	0

## Discussion

Diallel crosses allow estimation of the general combining ability (GC), which is associated with predominantly additive genes, and the specific combining ability (SCC), which is related to non-additive effect genes^13^. GCA was defined by Sprague and Tatum (1942) as the mean behavior of a parent line across a series of hybrid combinations, and this behavior results from the additive gene effect of the alleles. These authors defined the SCA as the vigor of a cross compared to that expected by the estimated GCA of the parents used in hybridization, which is determined by dominance genetic effects (complete or partial) and or epistasis.

SCA is interpreted as an additional effect on hybrid expression regarding the parental GCA effects, and can be positive or negative. SCA results from the interaction of parental GCA effects and can improve or worsen hybrid expression relative to the expected effect based on GCA alone^14^. SCA effects, estimated as deviation of behavior from what would be expected based on GCA, are measures of non-additive gene effects, those hybrid combinations with more favorable SCA estimates, involving at least one of the parents that showed the most favorable GCA effect, are desirable^15^.

We can note that the parents showed significant differences regarding the genotypes × environments interaction for most of the nutritional contents, which is one of the main challenges in the choice and recommendation of superior cultivars. This interaction allows the emergence of stable genotypes for specific environments or genotypes with general behavior adapted to a wide range of environments^16^. In this research, besides the distinct climatic factors (Fig. 2) of each location, the physical–chemical properties of the soil are important factors for the occurrence of significant genotype × environment interaction. Besides the need to obtain more productive cultivars, it is necessary to spend more on fertilizers. Adopting nutrient-efficient genotypes is a strategy, especially in cerrado soils, aiming to save costs and prevent environmental impacts.

When evaluating the nutrient contents, we found that the P content was significant for CEC, in which the parents G1, G5, and G7 and segregating populations P4, P5, P11, P14, P18, P19, P24, and P27 stand out. Thus, the difference between the means of the nutrients evaluated does not summarize only the individual behavior of the genitors^11,17^, but can also be attributed to environmental growing conditions. The soils of the cerrado are very weathered, with low levels of plant-available P, besides retaining nutrients in their colloids^18^. Under P limiting conditions, several metabolic problems can occur in the plants leading to delayed maturation and yield decrease^19^.

Obtaining information on the uptake and metabolization of P in plants allows the selection of these lines that have good development in soils with low P contents, in addition to makes it possible to use less phosphate fertilizers, which is essential for the sustainability of agricultural production aiming avoiding environmental problems caused by the incorrect use of fertilizers^20^. By studying P efficiency and responsiveness in soybean genotypes^21^, classified the cultivars studied into four groups: efficient and responsive, efficient and non-responsive, non-efficient and responsive, and non-efficient and non-responsive. The authors found that selecting P-use efficient cultivars in an environment with low availability of this nutrient favored the selection of cultivars responsive to the nutrient.

Using genotypes with a better capacity to accumulate potassium (K) contents results in improved carbohydrate and protein metabolism and starch translocation, which are used in grain formation^22^. Similarly, the selection of parents and segregating populations with higher Ca content provides enhanced structural metabolism, since the element acts in the cell wall synthesis, pollen tube growth, and pollen grain germination^23^. Genotypes more efficient in sulfur (S) uptake and metabolization, which has a structural and metabolic function in plants^24^ may be a promising strategy to develop cultivars better adapted to degraded soils, especially in the Brazilian Cerrado.

Mg content was significant for GCA, for which the genotypes G1, G3 and G7 and the segregating populations P1, P2, P20, P21, P24 and P27 stood out. Thus, at least one of the parents used in the crossings differed from the others regarding the concentration of alleles favorable for higher Mg expression^17^. The differential of obtaining lines with higher Mg contents in the plant is related to the role of this nutrient in activating enzymatic reactions and its presence in the structural part of plants (chlorophyll molecule)^25^.

In this context, the increasingly intensive use of soil, due to successive cropping, may result in degradations that lead to nutritional disorders in plants. Given this scenario, selecting genotypes containing higher levels of micronutrients is crucial for breeding programs. The parents G3 and G8 and the populations P7, P13, and P15 stood out for their relationship with micronutrients. Thus, selecting efficient genotypes in micronutrient uptake and metabolization makes the plant require a reduced amount of nutrients and have the same performance as the others to grow adequately in areas with nutrient limitations.

Besides improving plants' nutritional efficiency, soybean breeding programs have also aimed to develop cultivars combining higher earliness and grain yield^26,27^. According to Almeida et al., soybean cultivars can be classified as early (111 days), semi-early (112 to 124 days), and late (above 125 days). Soybean is a crop strongly influenced by weather conditions. By using early genotypes, farmers can minimize losses from end-of-cycle diseases^26,28^, besides allowing the growing of second-season maize^29^.

In the search for genotypes adapted to specific environments, the duration of the vegetative phase is an essential attribute to be considered. Plants that do not have juvenility genes will flower early, thus reducing the plant's size and leading to losses in grain yield^30^. Selecting early-flowering genotypes may lead to lower grain yield since these plants have a reduced height and number of nodes^31^.

Regarding DM, it is desirable that the genotypes present lower means (i.e., higher earliness), and that at least one of the parents has negative GCA estimates^32^. Parent G3 showed high negative GCA estimates for DM, suggesting a high concentration of alleles favorable to shortening the cycle of these soybean lines. The significant GCA effects indicate that some parents will contribute with a higher number of favorable alleles transmitted to the offspring^33^.

Considering the segregating populations evaluated in this experiment, P2, P9, P11, P12, P14, P23, P25, and P26 stood out for presenting negative specific combining ability in both locations evaluated. These findings reveal the possibility of obtaining earlier genotypes from these crossings after a few inbreeding generations^34^. However, among these populations, only P11, P12, P25, and P26 showed positive values for grain yield, with P11 and P26 showing the highest means.

Besides combining favorable traits such as nutritional efficiency, earliness and high yields, the current soybean cultivars must have improved levels for traits of industrial interest. With the increasing consumption of animal protein, the demand for bran for poultry, cattle and confined swine feed has been rising. To supply this sector, the industry needs soybean to have high grain protein and oil contents, which are at least 40% protein and 20% oil contents, while the national average protein is around 37%^35^. Early and indeterminate cycle cultivars tend to have higher protein contents, which may be a response to increased exposure to solar radiation and heat during the grain-filling phase^36^. By evaluating four soybean cultivars developed and improved in Brazil^37^, found protein contents ranging between 33.4% and 35.1%, values below those found here.

However, the increase in ash, fiber, oil and protein contents in soybean grains is a complex task for breeders, due to the high environmental influence on the genes^38^ and the existence of a negative relationship between these traits and with grain yield. For example, it is known that fiber levels have decreased over the years due to the increase in oil content, variables that are negatively correlated. Likewise, the oil content tends to decrease by selecting genotypes for higher protein content. Thus, the genotypes that stood out for the industrial variables should be used further in the breeding process, seeking to improve such traits simultaneously with the other characteristics of interest in the breeding pipeline.

The parents and segregating populations showed distinct responses for selecting nutritional, earliness, yield, and industrial traits. These genotypes should be monitored in the breeding process because they guide the breeders toward what they want to improve and attempt to achieve genotypes containing one or more traits of interest in a soybean cultivar.

Seeking to identify segregating parents and populations of soybean that get better characteristics regarding the uptake and metabolism of nutrients, earliness, yield, and contents of ash, fiber, oil and protein, our study aimed to identify this genetic variability between parents and populations through a diallel analysis. Our findings reveal that the parent G3 and the segregating populations P20 and P27 can be used for improved nutritional efficiency in new soybean cultivars. The segregating populations P11 and P26 show higher potential for selecting genotypes combining early maturity and high grain yield. The parent G5 and segregant population P6 are promising for selection seeking to improve industrial traits in soybean.

## Materials and methods

### Obtaining the progenies in the F_1_ generation

Hybrids were obtained in the greenhouse, using commercial cultivars as parents (Table 12). Divergence based on the relative maturity group (RMG) was considered as a selection criterion for the parents. Twenty-eight crossbreeds were performed to obtain the F_1_ generation, as described in Table 13. All methods were carried out in accordance with relevant guidelines with relevant institutional, national, and international guidelines and legislation.

**Table 12:** Characteristics of soybean cultivars used as parents.

Cultivar	Code	rmg	Flower color	Characteristics
Compacta	G1	6.5	Purple	High yield potential. Well-defined size with resistance to lodging. High branching potential
Lança	G2	5.8	White	High yield potential. Adaptation to higher altitude regions. Well-defined size with resistance to lodging. High branching potential
Zeus	G3	5.5	White	High yield potential. Earliness. Adaptation to higher altitude regions. Well-defined size with resistance to lodging
M6410	G4	6.4	Purple	High performance across different environments. Wide seeding period. Excellent plant branching and compensation
NS6909	G5	6.3	Purple	High potential and yield response in high-fertility soils. Potential to anticipate second-crop cultivation
TMG7061	G6	6.1	Purple	High yield. Earliness. Enables second-crop cultivation
TMG7062	G7	6.9	White	High yield. High grain weight
TMG7063	G8	7.0	White	High yield. High grain weight

**Table 13:** List of the twenty-eight F_1_ soybean populations obtained.

Population code	Male parent	Female parent
P1	Compacta	Lança
P2	Compacta	Zeus
P3	Compacta	M6410
P4	Compacta	NS6909
P5	Compacta	TMG7061
P6	Compacta	TMG7062
P7	Compacta	TMG7063
P8	Lança	Zeus
P9	Lança	M6410
P10	Lança	NS6909
P11	Lança	TMG7061
P12	Lança	TMG7062
P13	Lança	TMG7063
P14	Zeus	M6410
P15	Zeus	NS6909
P16	Zeus	TMG7061
P17	Zeus	TMG7062
P18	Zeus	TMG7063
P19	M6410	NS6909
P20	M6410	TMG7061
P21	M6410	TMG7062
P22	M6410	TMG7063
P23	NS6909	TMG7061
P24	NS6909	TMG7062
P25	NS6909	TMG7063
P26	TMG7061	TMG7062
P27	TMG7061	TMG7063
P28	TMG7062	TMG7063

### Obtaining the progenies in the F_2_ generation

Cultivation of the F_1_ hybrids was carried out in the greenhouse. The hybrids were sown in 3 L plastic pots (0.4 m of height and 0.3 m of width) and, after identification of the hybrid plants characterized by the purple color of the hypocotyl, one plant per pot was kept. Pest and disease control was performed according to technical recommendations for the crop using 2 L of soil in each pot.

### Conducting the F_2_ generation

**Figure 1:**
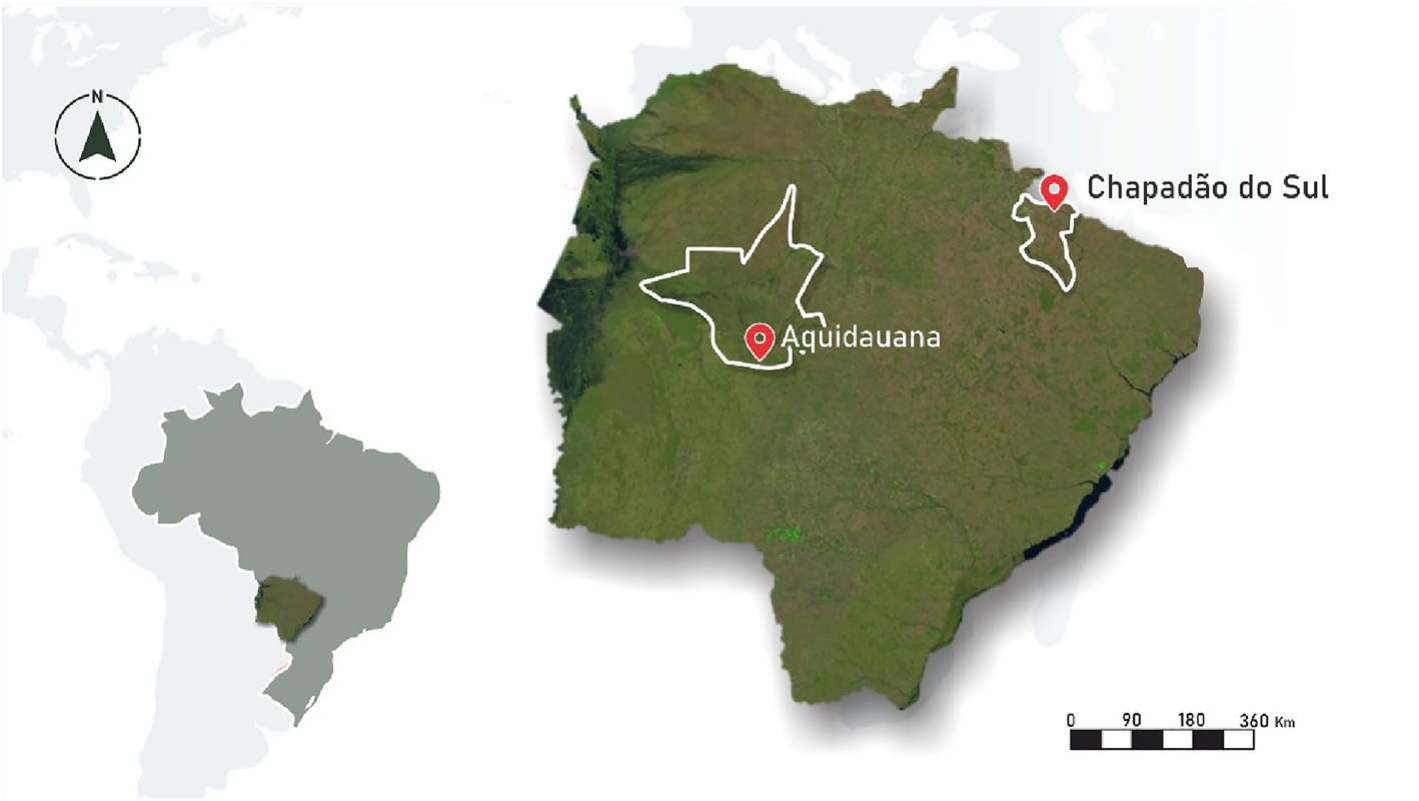
Location of experiments in the State of Mato Grosso do Sul (MS), Brazil.

Conduction of F_2_ populations was carried out in two locations: Aquidauana and Chapadão do Sul (Fig. 1). In the first site, the trial was installed at the State University of Mato Grosso do Sul, University Unit of Aquidauana (20°27′S, 55°48′W and average altitude of 120 m). The region's climate is Aw (Tropical Savanna) with mean annual rainfall of 1200 mm, and mean annual temperature of 24.2 °C.

In the second site, the trial was carried out at the experimental field of the Federal University of Mato Grosso do Sul, Chapadão do Sul Campus (18°46′S, 52°37′W and average altitude of 810 m). The region's climate is classified as Aw, with mean annual rainfall of 1850 m and mean annual temperature of 20.5 °C.

In Aquidauana, the soil of the experimental area was classified as a sandy-textured Red Dystrophic Argissolo, with the following chemical properties: pH (CaCl_2_) = 6.2; organic matter = 19.7 (g dm^−3^); P = 67.5 (mg dm^−3^); H + Al = 3.2; K = 32.0 (mg dm^−3^); Ca = 3.30 (cmolc dm^−3^); Mg = 2.10 (cmolc dm^−3^); cation exchange capacity (CEC) = 5.1 (cmolc dm^−3^); base saturation (V) = 45.0%.

The soil of the experimental area in Chapadão do Sul was classified as Red Dystrophic Latossolo, and has the following chemical properties: pH (CaCl_2_) = 4.8; organic matter = 17.6 (g dm^−3^); P = 5.0 (mg dm^−3^); H + Al = 5.3; K = 69.0 (mg dm^−3^); Ca = 1.6 (cmolc dm^−3^); Mg = 0.5 (cmolc dm^−3^); cation exchange capacity (CEC) = 7.6 (cmolc dm^−3^); base saturation (V) = 30.0%. Three months before sowing, liming was performed on the soil of both experimental areas to raise the base saturation to 60%.

In both locations, the experiments were implemented adopting a tillage system with one plowing and two harrowing (crusher and leveling harrows). Row opening and fertilization were mechanized with a five-row seeder spaced at 0.45 m between rows. The base fertilizer used was 300 kg ha^−1^ of the 04-14-08 NPK formulation. Seeding was performed manually by distributing 15 seeds per meter.

Seeds were treated with fungicide (Pyraclostrobin + Methyl Thiophanate) and insecticide (Fipronil), at a rate of 200 mL of the commercial product for every 100 kg of seeds to protect against the attack of pests and soil fungi. For biological nitrogen fixation (BNF), the seeds were inoculated with *Bradyrhizobium* spp. bacteria using a rate of 200 mL of concentrated liquid inoculant for every 100 kg of seeds.

**Figure 2:**
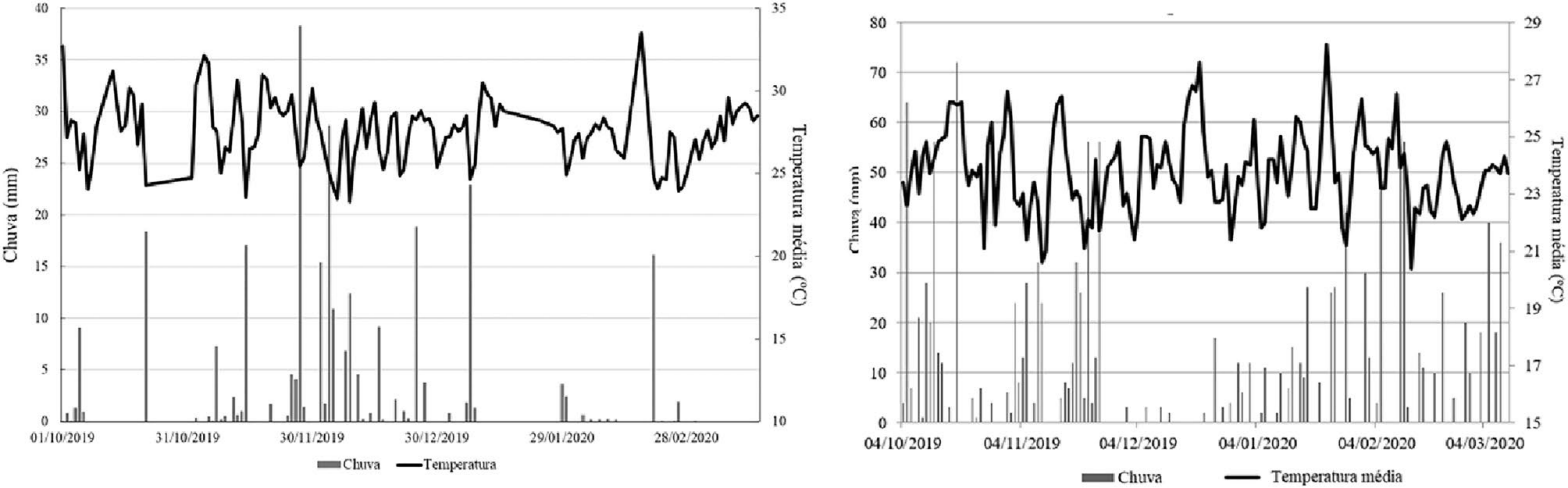
Weather conditions during the 2019/2020 crop season in the municipalities of Aquidauana (left) and Chapadão do Sul (right), MS, Brazil.

Crop management was performed according to the needs of the soybean crop. Figure 2 shows the weather conditions during the experiment.

### Experimental design and treatments

A randomized block design was used with two repetitions, eight parents (Table 1) and 28 F_2_ populations (Table 2). The plots consisted of one three-meter row, with 0.45 m spacing between rows and a density of 15 plants m^−1^. This size was adopted due to the limited quantity of seeds from the crosses carried out so that the genotypes could be evaluated in two locations.

### Traits evaluated in the F_2_ generation

At 60 days after emergence (DAE), the nutritional contents of phosphorus (P), potassium (K), calcium (Ca), magnesium (Mg), sulfur (S), copper (Cu), iron (Fe), manganese (Mn), and zinc (Zn) of each treatment were evaluated, following the methodology described in^39^.

For the nutritional analysis, we used the third fully developed leaf from the plant's apex, considered diagnostic for soybean nutritional analysis, where most metabolic processes responsible for energy acquisition occur. Twenty-five leaves with petioles were collected from each experimental unit. The nutritional contents of macronutrients were expressed in g kg^−1^, while micronutrients were expressed in mg kg^−1^.

Agronomic traits evaluated were: days to maturity (DM) and grain yield (GY, kg ha^−1^). DM corresponded to the days between emergence and maturation of more than 50% of plants in each experimental unit. GY was evaluated by harvesting the central 2 m of each plot and correcting for 13% moisture.

The measurement of protein (TP, %), total oil (TO, %), fiber (TF, %) and ash (TC, %) contents in F_2_ populations was performed by near-infrared spectroscopy (NIRS) (Metrohm, DS2500 spectrometer, Herisau, Switzerland) with high optical precision. Grain samples were homogenized and placed in a sampling dish. The analysis was based on illuminating a sample with a specific radiation wavelength in the near-infrared region and then measuring the difference between the amount of energy emitted by the spectroscope and reflected by the sample to the detector (AOAC, 2000). This difference was measured in several bands, creating a spectrum for each sample. The output was compared with a calibration set and expressed as a percentage.

### Statistical analyses

Initially, a joint analysis of variance was performed in Genes software according to the statistical model described below:

1 $$ {\text{Y}}_{{{\text{ijk}}}} =  \upmu  + {\text{B/A}}_{{{\text{jk}}}} + {\text{G}}_{{\text{i}}} + {\text{A}}_{{\text{j}}} + {\text{GxA}}_{{{\text{ij}}}} + {\text{e}}_{{{\text{ijk}}}} $$

wherein: Y_ijk_ is the observation in the k-th block, evaluated in the i-th genotype and j-th environment; µ is the overall mean of the experiments; B/E_jk_ is the effect of the block k within the environment j; G_i_ is the effect of the i-th genotype considered as fixed; A_j_ is the effect of the j-th environment taken as random; GxA_ij_ is the random effect of the interaction between genotype i and environment j; e_ijk_ is the random error associated with Y_ijk_.

Afterward, the unfolding of the genotype and the G × A interaction effects at each location was performed according to the partial diallel structure based on the progeny of F_2_ to obtain additive (gi) and dominance (sij) effects. Diallel analysis followed the model 4 proposed by Griffing^40^ to estimate general and specific combining abilities, as described below:

2 $$ {\text{Y}}_{{{\text{ij}}}} =  \upmu  + {\text{g}}_{{\text{i}}} + {\text{g}}_{{\text{j}}} + {\text{s}}_{{{\text{ij}}}} + {\text{e}}_{{{\text{ij}}}} $$

wherein: Y_ij_ is the mean of the crossbreeding between the i-th line from group 1 and the j-th line from group 2; µ is the overall mean of the diallel; g_i_ is the general combining ability of the i-th line from group 1; g_j_ is the general combining ability of the j-th line from group 2; s_ij_ is the specific combining ability between the lines from groups 1 and 2; e_ij_ is the mean experimental error.

Subsequently, grouping of means (overall across environments) of the 36 crossbreeds was performed by the Scott and Knott test at 5% probability level. All analyses were performed using the software Genes^41^, following the procedures recommended by Cruz et al.

### Supplementary Information

Supplementary Tables.

## Data Availability

The datasets used and/or analysed during the current study available from the corresponding author on reasonable request.
