# Supplementary material for: Understanding the combining ability of nutritional, agronomic and industrial traits in soybean F2 progenies
Source: Sci Rep. 2023 Oct 20;13:17909. doi: 10.1038/s41598-023-45271-4 (PMC10589319; doi:10.1038/s41598-023-45271-4)
Supplement: Supplementary file 1 — Supplementary Tables. [file 41598_2023_45271_MOESM1_ESM.docx]

**SUPPLEMENTARY FILES**

**Table S1.** Mean square of joint analysis of variance for the nutritional contents of P, K, Ca, Mg, S, Cu, Fe, Mn, and Zn assessed in leaf samples from parents and F_2_ segregating populations of soybean in two sites (Aquidauana and Chapadão do Sul, MS, Brazil)

| SV | DF | P | K | Ca | Mg | S | Cu | Fe | Mn | Zn |
| --- | --- | --- | --- | --- | --- | --- | --- | --- | --- | --- |
| Environments (E) | 1 | 6.70* | 1.08* | 80.32* | 5.32* | 3.52* | 31.17* | 34.31* | 73.44* | 251.22* |
| Block/E | 2 | 1.71* | 4.55* | 12.45* | 1.15* | 1.04* | 24.56* | 12.56* | 45.56* | 129.56* |
| Genotypes (G) | 35 | 0.14* | 8.43* | 6.19^ns^ | 0.93* | 0.09^ns^ | 34.16^ns^ | 5.99^ns^ | 64.07^ns^ | 133.23^ns^ |
| GCA | 5 | 0.10^ns^ | 7.90* | 11.31^ns^ | 0.75* | 0.18^ns^ | 21.18^ns^ | 8.08^ns^ | 59.62^ns^ | 225.56^ns^ |
| SCA | 25 | 0.15* | 8.56* | 4.90^ns^ | 0.98* | 0.07^ns^ | 37.41^ns^ | 15.47^ns^ | 65.18^ns^ | 110.15^ns^ |
| G x E | 35 | 0.07^ns^ | 8.07* | 3.93* | 0.41* | 0.14* | 19.62* | 16.28* | 124.30* | 109.79* |
| GCA x E | 5 | 0.03^ns^ | 8.86* | 5.44* | 0.17ns | 0.06^ns^ | 9.15ns | 24.18* | 146.93* | 82.19* |
| SCA x E | 25 | 0.08^ns^ | 7.87* | 3.55* | 0.47* | 0.17* | 22.23* | 14.31* | 118.64* | 116.69* |
| Error | 70 | 0.13 | 4.28 | 1.60 | 0.11 | 0.08 | 10.24 | 1.41 | 19.87 | 23.67 |
| Mean |  | ------- g kg^-1^ ------- | | | | | ------- mg kg^-1^ ------- | | | |
|  |  | 2.98 | 15.94 | 8.50 | 3.75 | 2.40 | 11.38 | 159.30 | 53.99 | 49.13 |
| CV (%) |  | 12.26 | 12.92 | 14.76 | 9.03 | 11.94 | 18.20 | 13.44 | 8.22 | 9.84 |

^ns^ and *: not significant and significant at 5% probability by the F test, respectively. SV: sources of variation; DF: degrees of freedom; GCA: general combining ability; SCA: specific combining ability. ^ns^ and *: non-significant and significant at 5% probability by the F-test, respectively.

**Table S2.** Mean square of joint analysis of variance for the agronomic traits days to maturity (DM) and grain yield (GY) assessed in leaf samples from parents and F_2_ segregating populations of soybean in two sites (Aquidauana and Chapadão do Sul, MS, Brazil).

| SV | GL | DM | GY |
| --- | --- | --- | --- |
| Environments (Env) | 1 | 164.69* | 57410171.30* |
| Block/E | 2 | 26.79* | 9875123.19* |
| Genotypes (G) | 35 | 12.43^ns^ | 1555641.84* |
| GCA | 5 | 11.16^ns^ | 1445373.92^ns^ |
| SCA | 25 | 12.75^ns^ | 1583208.82* |
| G x E | 35 | 9.89* | 601730.51^ns^ |
| GCA x E | 5 | 9.49* | 514270.77^ns^ |
| SCA x E | 25 | 9.99* | 623595.44^ns^ |
| Error | 70 | 2.49 | 772368.26 |
| Mean |  | 103.14 days | 2843.39 t ha^-1^ |
| CV (%) |  | 1.52 | 19.94 |

SV: sources of variation; DF: degrees of freedom; GCA: general combining ability; SCA: specific combining ability. ^ns^ and *: non-significant and significant at 5% probability by the F-test, respectively.

**Table S3.** Mean square joint analysis of variance for the industrial traits protein (PC), oil (OC), fiber (FC) and ash (AC) contents assessed in leaf samples from parents and F_2_ segregating populations of soybean in two sites (Aquidauana and Chapadão do Sul, MS, Brazil).

| SV | GL | PC | OC | FC | AC |
| --- | --- | --- | --- | --- | --- |
| Environments (Env) | 1 | 48.53* | 3.89* | 0.01^ns^ | 0.20* |
| Block/E | 2 | 23.44* | 12.25* | 0.01^ns^ | 0.18* |
| Genotypes (G) | 35 | 2.00* | 1.56* | 0.20^ns^ | 0.01^ns^ |
| GCA | 5 | 1.58^ns^ | 3.08* | 0.06^ns^ | 0.02^ns^ |
| SCA | 25 | 1.61^ns^ | 1.18* | 0.23^ns^ | 0.01^ns^ |
| G x E | 35 | 1.65* | 0.37* | 0.15* | 0.02* |
| GCA x E | 5 | 1.96* | 0.53* | 0.10* | 0.01^ns^ |
| SCA x E | 25 | 1.56* | 0.32* | 0.16* | 0.02* |
| Error | 70 | 0.27 | 0.09 | 0.03 | 0.01 |
| Mean |  | 36.80 | 21.34 | 5.51 | 4.99 |
| CV (%) |  | 1.46 | 1.43 | 3.54 | 1.50 |

SV: sources of variation; DF: degrees of freedom; GCA: general combining ability; SCA: specific combining ability; ^ns^ and *: non-significant and significant at 5% probability by the F-test, respectively.
